# Can intracellular cAMP dynamics enable scalable computation?

**DOI:** 10.3389/fncel.2015.00112

**Published:** 2015-03-27

**Authors:** Ravi Iyengar

**Affiliations:** Department of Pharmacology and Systems Therapeutics, Systems Biology Center, Icahn School of Medicine at Mount SinaiNew York, NY, USA

**Keywords:** cAMP, circuit, networks, adenylyl cyclases, computational modeling

cAMP, an important neuronal intracellular messenger, is produced in a spatially specified manner within neurons such that distinct patterns of elevated cAMP levels can be observed in dendrites. The spatial information encoded in local elevation of cAMP can be transmitted to downstream components such as MAP-kinases (Neves et al., 2008). Such spatially restricted changes in cAMP levels and information flow from cAMP to downstream effectors may enable it to function as a scaling agent for computation by signaling networks Computation within signaling networks may enable integration as well as sorting of signals from multiple receptors and channels. The output of signaling network computation can define thresholds for switching between states, temporal resolution of responses as well as other alterations to signal/response relationships. An intriguing question is whether such computation within signaling networks can be manifested in changes of electrical activity patterns of neuronal circuits.

The ability of cAMP to serve as an agent of scalability whereby computation within signaling networks can be manifested as altered patterns of circuit activity arising from two other major factors: (a) The production of cAMP is controlled by the expression of multiple adenylyl cyclases within a neuron that integrate signals from different receptor types and channels in a mix and match format (Pieroni et al., 1993). (b) cAMP has its effects through multiple effectors. These include protein kinase A, the cAMP-dependent GEFs and the cyclic nucleotide gated channels (HCN channels). The multiplicity of adenylyl cyclases provides (Pieroni et al., 1993; Jordan et al., 2000) for a rich capability to compute relationships between different input signals and have this computation reflected in net changes in local cAMP levels. (Figure 1A upper part) The multiplicity of effectors ensures that cAMP dynamics can be captured across different time scales from acute electrophysiological effects mediated by the HCN channels to medium term effects mediated by regulation of phosphorylation of other channels and longer term effects such as changes in gene expression mediated by PKA or by cAMP GEFs that can through Rap regulate the activity of MAP-kinases (Figure 1A lower). Regulation of gene expression by both PKA and MAP-kinases enables cells to alter the levels of numerous components within these signaling networks and thus can further enhance the computational capability of the cAMP signaling network in different regions of the neurons.

**Figure 1:**
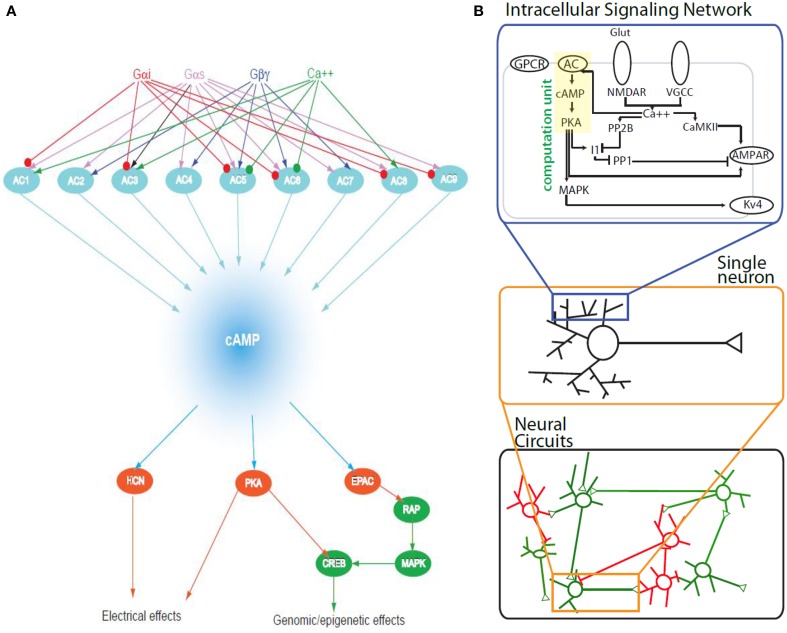
**(A)** The cAMP signaling network organized for computing. In this simplified bow-tie diagram the ability of different adenylyl cyclases to receive signals from different G proteins that couple to different types of receptors as well as from calcium is shown as the upper half. Integration of these signals can be reflected in the levels of cAMP which may represent different types of computation such as addition, subtraction or multiplication. The bottom half of the bowtie shows effectors of cAMP that include ion channels, protein kinases and guanine nucleotide exchange factors such as EPAC that respond to changes in cAMP levels and thus change cellular responses at different time spaces. **(B)** A schematic representation of the scaling of computation within the cAMP signaling network. Upper panel represents the cAMP cell signaling network within spines and dendrites. Computation within such a single network can alter the ability of a neuron to display a firing pattern (middle panel). When such a neuron is part of a circuit it can alter the electrical activity of the circuit that in turn can result in change in organismal behavior.

The role of the direct binding of cAMP to HCNs and regulating the activity of these channels is particularly relevant for its ability to act as a scaling agent. HCN channels are hyperpolarizing and are activated by a combination of membrane depolarization and cAMP binding. Local changes in cAMP levels in dendrites coupled with differential distribution of HCNs that are localized in the distal dendrites in pyramidal neurons (Magee, 1998) can regulate excitability in the hippocampus. In other types of neurons the HCN channels are localized to other regions within the neurons (Magee, 1998) and can thus regulate differing types of electrophysiological responses. In addition to directly controlling the HCN channels, cAMP, through protein kinase A regulates the insertion of AMPA channels into the postsynaptic region thus regulating synaptic excitability. This regulation is controlled by a dense network involving both phosphodiesterases as well as MAP-kinases (Song et al., 2013) thus allowing for additional computational capability depending on both transcriptional and translational control. PKA also controls NMDA-type channels and voltage gated calcium channels by phosphorylation (Gray et al., 1998). Taken together, cAMP regulation of all these channels allows for a varied expression of electrophysiological responses of individual neurons in response to changes in cAMP levels in different subdomains. Since the specialized shape of the neuron can effectively restrict changes in cAMP levels to different regions the information from cell shape such as dendritic arborization can be coupled to electrophysiological activity through the local levels of cAMP. Further since the net level of cAMP is reflective of the computation that occurs due to the presence of multiple adenylyl cyclase isoforms and their differential regulation it can be readily seen how computation within signaling networks can be expressed as altered electrophysiological responses in individual neurons.

For the relationship between local cAMP levels and the overall electrophysiological response of the neuron to be scalable, the altered excitability of the individual neuron needs to be reflected in the overall electrical behavior of the circuit of which the neuron is a part. At this level there is little experimental data that would allow us to build specific conjectures that one can turn into experimental or computationally testable hypothesis. For this, two classes of data are needed anatomical/physiological connectivity between neurons and biochemical activity in individual cells as part of a functional tissue.

The first class of data that is part anatomical and part electrophysiological is being called connectomics. We need to get a first–pass description of the organization of the circuit at the level of individual neurons and their connections. So far this is only known in the worm (Jarrell et al., 2012), which has only 302 neurons (White et al., 1986). We need to identify how the excitatory neurons, inhibitory interneurons and glial cells are connected to one another within a functional circuit, and determine that the anatomically observed synapses are electrically active. We also need to know how the modulatory inputs into the circuit are organized since modulatory regulation, such as those by adrenergic receptors, play a major role in tuning neuronal function at a cellular level. Furthermore, we need to identify the regulatory network motifs such as both feedfoward paths through interneurons as well as short and long range feedback loops if they exist. Even for an extensively studied region such as the CA3-CA1 region of the hippocampus detailed circuit topology is not yet available for rodents or humans. Hopefully as the ambitious BRAIN project takes shape newer technologies will help gain such knowledge.

The second class of data we need is measurements of biochemical activities in individual cells when cells are part of functional circuitries and how such biochemical activity is related to cellular electrophysiology. Initially such cell-based measurements within tissue may be conducted *in vitro* such as in brain slices but eventually we are going to need information regarding biochemical activities at the cellular level *in vivo*. The cAMP network can serve as a prototype for such studies since the cAMP live cell imaging probes that are used in cultured neuron experiments also work in the tissues (Castro et al., 2010). Studies of cAMP levels in hippocampal slices (Castro et al., 2010) are in agreement with computational predictions developed using kinetic parameters estimated from purified proteins (Neves et al., 2008). This type of quantitative convergence indicates that we should be able to develop computational models that can make useful predictions regarding regulation of signal flow within intracellular signaling networks controlling electrical output. A recent study using live cell imaging of cAMP dynamics in mouse tissue nicely demonstrates the potential for pathway analysis and study of physiological responses (Castro et al., 2013).

If the experimental technologies are developed to get connectomics data in the various brain regions and imaging advances allow us to follow biochemical activities of one or a few neurons as they function within tissue, in combination with multielectrode recording of circuits, then we should be able to obtain scalable information of how and when computation within signaling networks are manifested in changes in circuit behavior. Such studies beyond providing fundamental understanding of information processing in the brain may help us understanding ageing as well as pathophysiology induced cognitive decline. A number of components of the cAMP signaling pathways and cAMP regulated genes such as BDNF change with age and such changes are associated with cognitive deficits both in normal ageing and pathophysiology such as Alzheimer's disease (Hansen and Zhang, 2013). One hypothesis could be that reduced capability of biochemical computations when transmitted to circuit level functions results in observed cognitive deficit. Alternatively, damage to circuit connectivity could result in inability of the circuit to convert biochemical computation in into altered organismal behaviors. So from both basic knowledge and disease mechanism viewpoints a question of particular interest for me is what circuit configurations at the multicellular level (i.e., connectivity between neurons) enable explicit manifestation of biochemical computation within signaling networks as functionally altered electrophysiological responses of neuronal circuits that in turn evoke different behaviors in the intact animals. Some circuit configurations may enable computations within signaling networks to be manifested as organismal behaviors while with other circuit configurations computation at the circuit level may drive behavioral responses. Current efforts such as the BRAIN project indicate that we should be able to design and conduct such experiments and build computational models to understand such multiscale functions. (Figure 1B) Here too studies on the cAMP systems could lead the way as it did when we started to move from pathways to networks (Pieroni et al., 1993; Iyengar, 1996).

## Conflict of interest statement

The authors declare that the research was conducted in the absence of any commercial or financial relationships that could be construed as a potential conflict of interest.
